# Radiomics Signatures of Computed Tomography Imaging for Predicting Risk Categorization and Clinical Stage of Thymomas

**DOI:** 10.1155/2019/3616852

**Published:** 2019-05-28

**Authors:** Xihai Wang, Wei Sun, Hongyuan Liang, Xiaonan Mao, Zaiming Lu

**Affiliations:** Department of Radiology, Shengjing Hospital, China Medical University, Shenyang 110000, China

## Abstract

**Purpose:**

The aim of this study is to develop and compare performance of radiomics signatures using texture features extracted from noncontrast enhanced CT (NECT) and contrast enhanced CT (CECT) images for preoperative predicting risk categorization and clinical stage of thymomas.

**Materials and Methods:**

Between January 2010 and October 2018, 199 patients with surgical resection and histopathologically confirmed thymoma were enrolled in this retrospective study. We extracted 841 radiomics features separately from volume of interest (VOI) in NECT and CECT images. The features with poor reproducibility and highly redundancy were removed. Then a least absolute shrinkage and selection operator method (LASSO) logistic regression model with 10-fold cross validation was used for further feature selection and radiomics signatures build. The predictive performances of radiomics signatures were assessed by receiver operating characteristic (ROC) analysis. The areas under the receiver operating characteristic curve (AUC) between radiomics signatures were compared by using Delong test.

**Result:**

In differentiating high risk thymomas from low risk thymomas, the AUC, sensitivity, and specificity were 0.801(95% CI 0.740–0.863), 0.752 and 0.767 for radiomics signature based on NECT images, and 0.827 (95% CI 0.771 -0.884), 0.798, and 0.722 for radiomics signature based on CECT images. But there was no significant difference (p=0.365) between them. In differentiating advanced stage thymomas from early stage thymomas, the AUC, sensitivity, and specificity were 0.829 (95%CI 0.757-0.900), 0.712, and 0.806 for radiomics signature based on NECT images and 0.860 (95%CI 0.803-0.917), 0.699, and 0.889 for radiomics signature based on CECT images. There was no significant difference (p=0.069) between them. The accuracy was 0.819 for radiomics signature based on NECT images, 0.869 for radiomics signature based on CECT images, and 0.779 for radiologists. Both radiomics signatures had a better performance than radiologists. But there was significant difference (p = 0.025) only between CECT radiomics signature and radiologists.

**Conclusion:**

Radiomics signatures based on texture analysis from NECT and CECT images could be utilized as noninvasive biomarkers for differentiating high risk thymomas from low risk thymomas and advanced stage thymomas from early stage thymoma. As a quantitative method, radiomics signature can provide complementary diagnostic information and help to plan personalized treatment for patients with thymomas.

## 1. Introduction

Thymomas are the most common primary neoplasms of anterior mediastinal masses, accounting for 47% of mediastinal neoplasms [1]. WHO classification which was proposed in 1999 classified thymomas into five types (A, AB, B1, B2, and B3) based on the morphology of epithelial cells as well as the lymphocyte-to-epithelial cell ratio [2, 3]. The Masaoka staging system based on anatomic extent of tumor and microscopic invasive properties of the tumor on surgical resection is the most widely used system in clinical practice [4]. These two systems have an important implication in determining treatment strategies and are considered to be independent prognostic factors [5–8].

According to previous study, type B2 and type B3 thymomas had more invasive behavior compared with types A, AB, and B1. And also patients with type B2 and type B3 thymomas had higher tumor recurrence rate and lower survival rate than patients with types A, AB, and B1 [7]. Thymomas of type A, AB, or B1 had more chances to be completely resected than type B2 or B3 [8]. Therefore many studies divided thymomas into low risk group (type A, type AB, and type B1) and high risk group (type B2, type B3) [9, 10]. The advanced stage of thymoma invades into adjacent organs and vessels. The early stage thymoma only spreads into fat surrounding the thymus or mediastinal pleura [4]. Postoperative classification Masaoka stage of thymoma is very important for evaluating surgical risk. Some previous study indicated that neoadjuvant therapy provided a survival benefit for patients with stage III thymomas [10–13]. Thus, postoperative prediction of Masaoka stage of thymoma guides decisions about neoadjuvant therapy.

Radiomics based on the high-dimensional quantitative features extracted from CT or MR imaging data can noninvasively quantify tumor heterogeneity and show underlying malignant features [14, 15]. Radiomics models were used to classify tumor stage and predict lymph node metastasis and prognosis [16–29]. Although previous studies demonstrated that texture analysis based on CT images could differentiate high risk thymomas from low risk thymomas, only 2D texture features were analyzed and the sample sizes were small [30, 31]. Our study focused on building radiomic signatures based on 3D texture analysis to differentiate high risk thymomas from low risk thymomas and advanced stage thymomas from early stage thymomas. We also compared the predictive performance between radiomic signatures based on NECT and CECT images.

## 2. Materials and Methods

### 2.1. Patients

**Figure 1:**
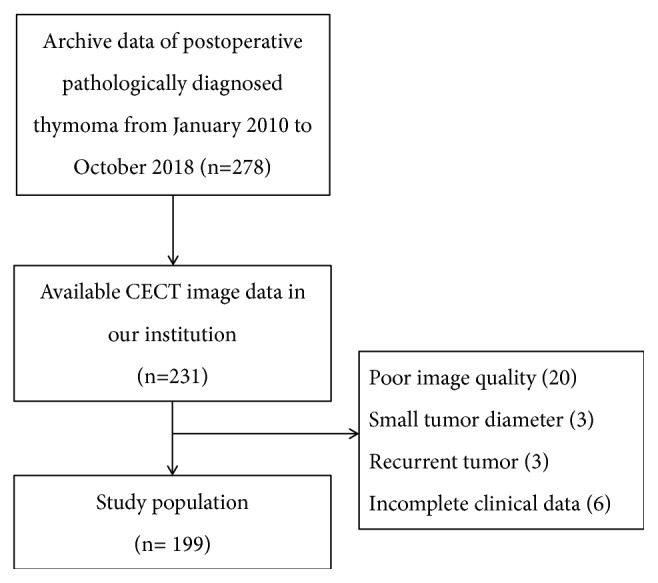
Patients selection flow diagram.

This retrospective analysis was approved by the Ethics Review Board. The need to obtain informed consent was waived. Patients who underwent surgical resection between January 2010 and October 2018 with pathologically confirmed thymomas were retrospectively retrieved in our institution. Inclusion criteria were as follows: (1) underwent tumor resection and pathologically diagnosed thymoma; (2) no previous treatment before CT scan; (3) underwent contrast enhanced CT imaging within two weeks before surgery; (4) available for clinical data and surgical record. Exclusion criteria were: (1) small tumor diameter (longest diameter < 9 mm); (2) poor image quality due to artifacts or other reasons. Finally a total of 199 patients were enrolled in our study and 79 patients were excluded (Figure 1). The Masaoka clinical stage and WHO histologic classification of thymomas were confirmed by reviewing the surgical findings and pathological examinations.

### 2.2. Image Acquisition

All patients underwent chest CT scans before and after intravenous administration of iodinated contrast agent (Visipaque 320, Amersham Health, Cork, Ireland) with 64-MDCT (Defnition, Siemens Healthcare, Erlangen, Germany), 128-MDCT (iCT, Philips Healthcare, Amsterdam, Netherlands), or 320-MDCT (Aquilion One, Toshiba Medical Systems Corp., Tokyo, Japan). The CT scans were acquired with following clinical protocol: 3 mm slice thickness, reconstruction interval 3 mm, tube voltage 100-120 kV and tube current 80-300 mA, high-resolution matrix size 512 × 512, and FOV 500 mm. A total of 80 mL of contrast material had been administered by an antecubital vein at a rate of 2.5 ml/s. The contrast enhanced CT scans were performed with 30s delay.

### 2.3. Segmentation

**Figure 2:**
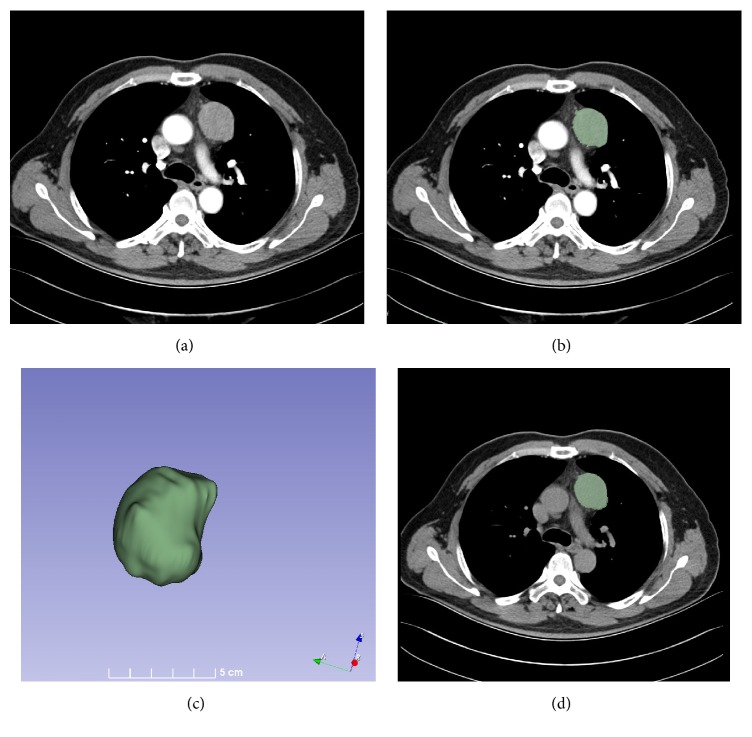
The process of segmentation. (a) Enhanced axial CT scan revealed the presence of a round mass in the left anterior mediastinum. (b) VOIs were drawn in all axial CT images. (c) VOI masks extracted from CT scans. (d) VOI masks were applied to NECT images.

Segmentations of entire tumor were performed by two experienced radiologist (S.W. and Z.M.L.; reader 1 and 2, with more than 10 years of experience in chest CT study interpretation, respectively) who were blind for pathology results with 3D Slicer software (version 4.10, www.slicer.org) [32]. The segmentations of VOI were separately performed in 40 randomly chosen images by both readers and interobserver reproducibility of texture feature was analyzed. The segmentations for the other images were completed by reader 1. To segment entire volume of tumor in all axial CT images, we took a method combining semi-automated and manual segmentation together. Firstly, we used threshold tool to determine a threshold range (0HU-140HU) and saved results to selected segmentation. Secondly we manually separated the lesion from the large blood vessels and chest wall. Finally, we used identify islands tool to create a unique segmentation. The VOI masks outlined in CECT images were applied to NECT images. Sometimes we redrew the VOI in NECT images due to movement of heart and lung. The process of segmentation took about 30 minutes for each patient and was displayed in Figure 2.

### 2.4. Image Feature Extraction

Extractions of radiomics features from VOIs were performed by using an extension of 3D Slicer software called SlicerRadiomics (V2.10, http://download.slicer.org.) [33]. SlicerRadiomics encapsulated with pyradiomics library is an extension of 3D Slicer software and can calculate a variety of radiomics features. The extension applies wavelet filter to VOIs and yields 8 derived images. Radiomics features are subdivided into the following classes:

  First-Order Statistics (18 features)
  Shape-Based (13 features)
  Gray Level Co-Occurence Matrix (23 features)
  Gray Level Run Length Matrix (16 features)
  Gray Level Size Zone Matrix (16 features)
  Neigbouring Gray Tone Difference Matrix (5 features)
  Gray Level Dependence Matrix (14 features)

Radiomics features per VOI included 13 shape descriptors and 828 features extracted from original and 8 derived images obtained by applying Wavelet filters. A total of 841 radiomics features were separately extracted from NECT and CECT VOIs for each patient. The details of radiomics features were described in supplementary data (available here).

### 2.5. Clinical Staging by Radiologist Interpretation

Two chest radiologists (W.S. and Z.M.L., with more than 10 years of experience in chest CT study interpretation, respectively) who were blind to the histologic classification and clinical information reviewed the chest CT scans. Decisions concerning the tumor stage were reached by consensus. The tumor stage was evaluated by radiologist based on the presence of mediastinal fat infiltration, pleural and pericardial effusion, invasion of the great vessels, pleural metastases, lymph node enlargement (short-axis diameter > 10 mm), and metastases [34].

### 2.6. Statistical Analysis

All statistical analyses were performed using R software (version 3.5.1, https://www.r-project.org/). The LASSO regression model and ROC curve analysis were conducted based on “glmnet” and “pROC” packages, respectively.

Radiomics feature selection was separately done in 841 features extracted from NECT and CECT VOIs. To improve predictive performance of model and avoid overfitting, dimension reduction was performed based on reproducibility and redundancy. Firstly, the ICC values of each feature were calculated to evaluate the interobserver reproducibility. Only the features with ICC value ≥0.9 were selected for further analysis. Secondly, we used Pearson's correlation matrix method to eliminate redundant features. The correlation coefficient between each feature was calculated and the features with correlation coefficient ≥0.9 were removed until there was no correlation coefficient ≥0.9 in correlation matrix. The features selected by above two steps were applied to LASSO logistic regression model after standardized.

A LASSO logistic regression model with 10-fold cross-validation was used to further select radiomics features and build radiomics signatures. LASSO logistic regression model was introduced to improve the prediction accuracy and interpretability of regression models by altering the model fitting process to select only a subset of the provided covariates for use in the final model rather than using all of them [35]. Radiomics signatures were calculated from selected features weighted by their regression coefficients for each patient.

Radiomics signatures between groups (low risk group and high risk group, advanced stage group, and early stage group) were tested by Mann–Whitney U test. Potential predictive performance of radiomics signatures was evaluated by ROC analysis. The AUC, accuracy, sensitivity, and specificity of radiomics signatures were calculated at cutoff point of Youden index (the highest sum of sensitivity plus specificity). A technique of bootstrapping (2000 samples) was used for internal validation. The AUCs of radiomics signature between NECT images and CECT images were compared by using Delong test [36]. The accuracy between radiomics signatures and radiologists' interpretation was compared by using Chi-square test.

## 3. Result

### 3.1. Patients Characteristics

Of all the patients, 80 patients were male, 119 patients were female. The age of patients ranged from 30 to 80 years. Thoracoscopic or thoracoscopic assisted thymectomies were performed in 144 patients and other patients underwent thoracotomies. The clinical and histopathology data of patients was showed in Table 1. There were 109 patients (19 type A, 44 type AB, and 46 type B1) with low risk thymomas and 90 patients (68 type B2, 22 type B3) with high risk thymomas. The Masaoka clinical stage was stage I in 123 patients, stage II in 41 patients, stage III in 27 patients, and stage IV in 8 patients. There were 164 patients with early stage thymomas, 35 patients with advanced thymomas. 72 patients were asymptomatic. The most common symptoms were myasthenia gravis in 47 patients, followed by chest comfort or pain in 31 patients, cough or dyspnea in 30 patients, and other reasons in 19 patients.

### 3.2. Radiomics Signature Building

**Figure 3:**
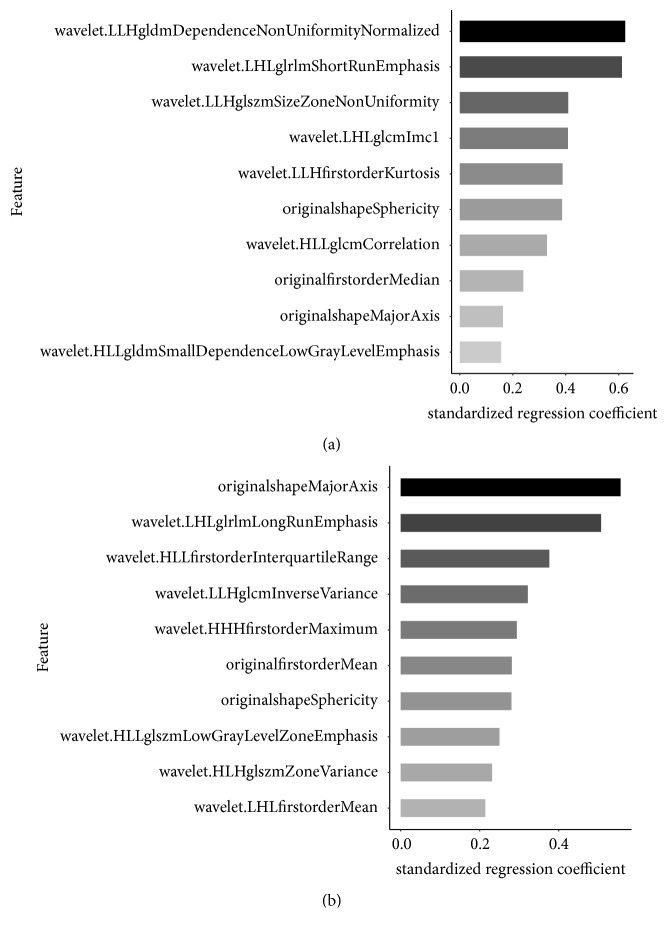
The top 10 features contributed to radiomics signatures based on NECT images (a) and CECT images (b) weighted by standardized regression coefficients according to LASSO logistic regression model to differentiate high risk thymomas from low risk thymomas.

For differentiating high risk thymomas from low risk thymomas, two independent radiomics signatures were built separately from NECT and CECT images. LASSO model based on NECT images selected 24 radiomics features and 34 radiomics features were selected for CECT model. The top 10 features contributed to radiomics signature weighted by standardized regression coefficient were displayed on Figure 3.

**Figure 4:**
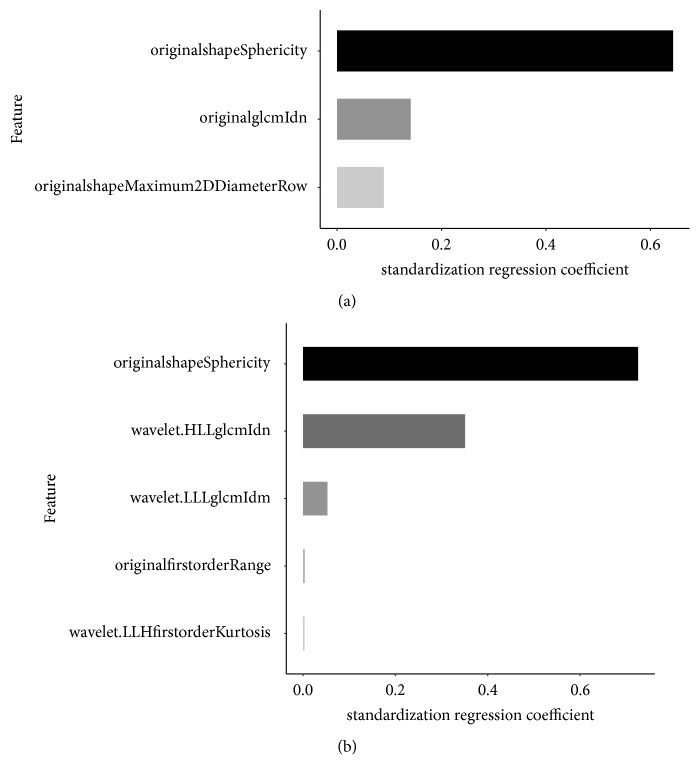
Attributing weights of radiomics features based on NECT images (a) and CECT images (b) selected by LASSO model to differentiate advanced thymomas from early thymomas.

The other two independent radiomics models were built separately from NECT and CECT images to differentiate advanced stage thymomas from early stage thymomas. LASSO model selected 3 radiomics features to build radiomics signature based on NECT images and 5 radiomics features to build radiomics signature based on CECT images. The contributions of radiomics signature weighted by standardized regression coefficient were displayed on Figure 4. The radiomics feature of “orginalshapSphericity” was the most significant affecting factor for both NECT and CECT radiomics signatures.

### 3.3. Predictive Performance of the Radiomics Signature

**Figure 5:**
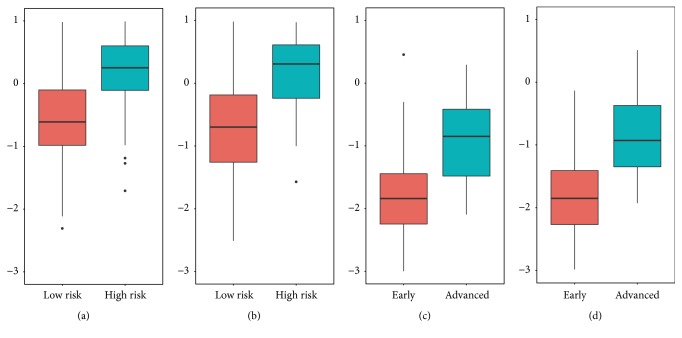
(a) A boxplot shows difference between low risk thymomas and high risk thymomas in radiomics signature based on NECT images. (b) A boxplot shows difference between low risk and high risk thymomas, in radiomics signature based on CECT images. (c) A boxplot shows difference between advanced stage and early stage thymomas, in radiomics signature based on NECT images. (d) A boxplot shows difference between advanced stage and early stage thymomas, in radiomics signature based on CECT images.

**Figure 6:**
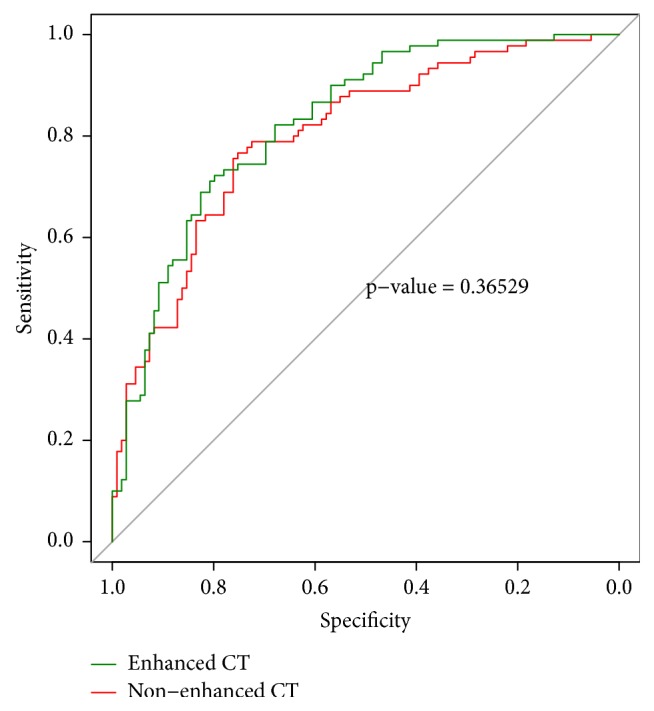
ROC curves analysis of radiomics signatures based on CECT images and NECT images for differentiating high risk thymomas from low risk thymomas.

There were significant difference between groups (low risk and high risk groups, advanced stage, and early stage groups) in radiomics signatures (P<0.05) (Figure 5). In discriminating high risk thymomas from low risk thymomas, the AUCs were 0.801 (95% CI 0.740–0.863) for radiomics signature based on NECT images and 0.827 (95% CI 0.771 -0.884) for radiomics signature based on CECT images. The sensitivity and specificity were 0.752 and 0.767 at optimal cutoff value (-0.104) for radiomics signature based on NECT images, 0.798 and 0.722 at optimal cut-off value (-0.110) for radiomics signature based on CECT images. However there was no significant difference between radiomics signatures based on CECT and NECT images (p=0.365) to discriminate high risk thymomas from low risk thymomas (Figure 6).

**Figure 7:**
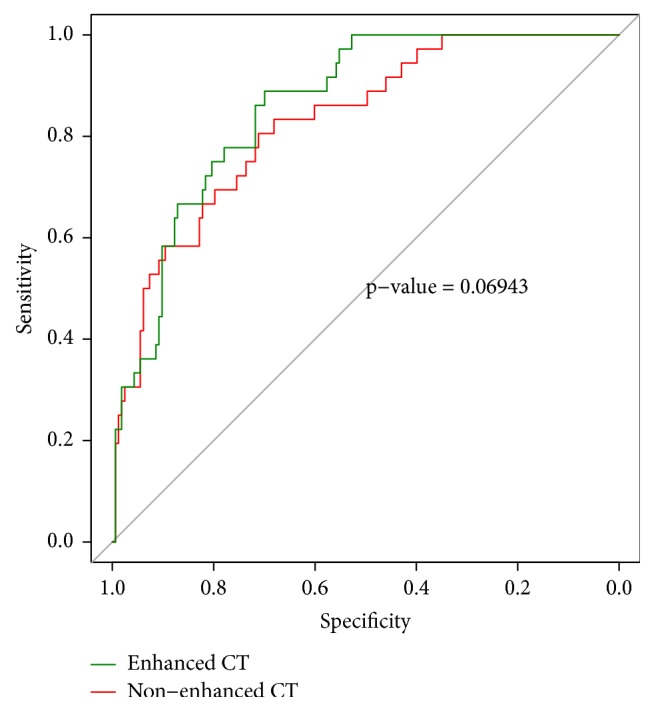
ROC curves analysis of radiomics signatures for differentiating advanced thymoma from early stage thymomas.

In differentiating advanced stage thymomas from early stage thymomas, AUC, sensitivity, and specificity for radiomics signature based on NECT images were 0.829 (95%CI 0.757-0.900), 0.712 and 0.806 (optimal cut-off value was -1.534), 0.860 (95%CI 0.803-0.917), 0.699 and 0.889 (optimal cut-off value was -1.545) for radiomics signature based on CECT images. There was no significant difference between CECT and NECT radiomics signatures (p=0.069) (Figure 7). The accuracy of radiomics signatures was 0.819 for NECT, 0.869 for CECT, and 0.779 for radiologists. Radiomics signatures had a better performance than radiologists' interpretation. But there was significant difference (p= 0.025) only between CECT radiomics signature and radiologists.

## 4. Discussion

The WHO classification and Masaoka clinical stage were predictive factors for recurrence and overall survival for patients with thymomas [5–8]. Preoperative prediction of histologic subtypes and clinical stage of thymomas can help to plan personalized treatment. Our study demonstrated that radiomics signatures based on NECT and CECT images had a good predictive performance in distinguishing high risk thymomas from low risk thymomas and advanced stage thymomas from early stage thymomas.

Some previous study attempted to differentiate high risk thymomas from low risk thymomas [30, 31]. Yasaka K et al. built radiomics model by logistic regression analysis and obtained high diagnostic performance. The AUCs for differentiating high risk thymomas from low risk thymomas was 0.89 for mean0c and 0.87 for combination of mean0u and entropy6u [31]. However in our study, the AUCs of radiomics signatures were 0.83 for CECT radiomics signature, 0.80 for NECT radiomics signature, which were lower than previous study. We thought the different radiomics features extracted from 2D or 3D texture analysis and the different classifiers were the main reasons that caused the difference. In our study, high-dimensional radiomics features up to 841 features were obtained. The feature engineering was very important for high-dimensional radiomics features to avoid overfitting. The features with poor reproducibility and highly redundancy were removed in our study, which might affect the AUCs of radiomics signatures. Finally, only a small amount of features were selected to build regression model. In previous study, the sample size would influence construction of reliable logistic regression models.

Recently radiomics was used to differentiate malignant tumors from benign tumors, predict prognosis and clinical staging of tumor, which attracted considerable attention [16–29]. Previous studies demonstrated that presence of contour, capsule, septum, and homogenous enhancement was helpful to distinguish low-risk thymomas from high-risk thymomas and carcinomas [9, 34, 37]. Abdel Razek AA et al. reported that significant difference was found between high risk thymomas and low risk thymomas in ADC values obtained from diffusion weighted MR imaging [10]. In our study we only included patients with thymomas and excluded patients with thymic carcinomas (type C). Histological heterogeneity between thymomas and thymic carcinomas could affect texture analysis. Previous study demonstrated that high risk thymomas are more heterogeneous compared with low risk thymomas. Many studies confirmed that proteogenomic and phenotypic information could be predicted by texture analysis [38, 39]. Radiomics signatures based on NECT and CECT images have almost same predictive performance in classifying the risk of thymomas. The NECT scan is routinely performed for every patient in clinic, and radiomics signature can easily be calculated based on NECT images.

The NECT and CECT radiomics signatures obtained similar predictive performance in differentiating advanced stage thymomas from early stage thymomas. However both radiomics signatures obtained higher accuracy than radiologist interpretation. Previous studies proved a close relationship between preoperative CT thymoma staging and postoperative Masaoka clinical staging. Although the weighted kappa coefficient was 0.819, which represented a strong consistency between CT stage and clinical stage [40], the accuracy of four clinical CT stage was only 0.68. We believed that the accuracy would greatly improve if binary classifications were used instead of four classifications. The radiomics model can be used to discriminate advanced stage from early stage thymomas and it can provide complementary diagnostic information for patients with thymomas.

Several studies showed an improvement in classification accuracy when using 3D texture analysis compared with 2D texture analysis [25, 41]. Previous study demonstrated 3D texture analysis showed 12% improvement in AUC and 19% in overall classification accuracy compared with 2D texture analysis in classification of childhood brain tumors [41]. Texture analysis based on one slice might not be sufficient to build a reliable classification model, because the features presenting heterogeneities across the tumor volume would not be included in model. And also 3D texture analysis could be able to capture inter-slice features that were completely ignored in the traditional 2D approach. However the 3D segmentation of the lesion is more complex and time-consuming than 2D segmentation. Further study would be needed to compared 3D and 2D texture analysis in classification the risk of thymomas.

Our study had several limitations. First, sample size between advanced and early stage patients was imbalanced. We evaluated predictive performance of radiomics signatures by ROC curves analysis, which was unaffected by imbalanced sample size. However in order to evaluated model proposed in our study, databases with balanced sample size are still needed. Secondly, all of the CT scans were performed in one single clinical center by 3 different CT scanners. Different CT scanners have significant difference parameters in CT scanning and reconstruction algorithm, which can affect texture analysis [42]. Thirdly, overfitting is an important problem in machine learning when dealing with high-dimensional features with small sample size. In our study 841 texture features were calculated and sample size was 199, so we attempted to mitigate overfitting by using 10-fold cross-validation. Fourthly, the TNM staging system adopted by the Union for International Cancer Control (UICC) in 2016 for thymic epithelial tumors was not used in our study [43]. Further study will be needed to reveal the relationship with texture feature and TNM staging system.

## 5. Conclusions

Radiomics signatures based on texture analysis extracted from NECT and CECT scan could be utilized as noninvasive biomarkers for differentiating high risk thymomas from low risk thymomas and advanced stage thymomas from early stage thymoma. As a quantitative method, radiomics signature can provide complementary diagnostic information and help to plan personalized treatment for patients with thymomas.

## Figures and Tables

**Table 1:** Characteristics of patients and tumors.

Characteristic	Patients
*Sex*	
Male	80
Female	119
*WHO Classification*	
A	19
AB	44
B1	46
B2	68
B3	22
*Masaoka Staging*	
Stage I	123
Stage II	41
Stage III	27
Stage IV	8
*Symptom*	
No	72
Myasthenia gravis	47
Chest comfort or pain	31
Cough or dyspnea	30
Other reasons	10
*Surgical Treatment*	
Thoracoscopic thymectomy	144
Thoracotomy	55
*CT scans*	
iCT	98
Definition64	34
Aquilion/640	67

## Data Availability

The R code used to support the findings of this study is available from the corresponding author upon request.

## Supplementary Materials

Supplementary Materials The supplementary material contains various radiomics features that can be extracted by using “PyRadiomics” in our study. The details of radiomics features are described in https://pyradiomics.readthedocs.io/en/latest/index.html.
