# Supplementary material for: Radiomics Signatures of Computed Tomography Imaging for Predicting Risk Categorization and Clinical Stage of Thymomas
Source: Biomed Res Int. 2019 May 28;2019:3616852. doi: 10.1155/2019/3616852 (PMC6558631; doi:10.1155/2019/3616852)
Supplement: Supplementary Materials — The supplementary material contains various radiomics features that can be extracted by using “PyRadiomics” in our study. The details of radiomics features are described in https://pyradiomics.readthedocs.io/en/latest/index.html. [file 3616852.f1.docx]

Radiomics Features

The radiomics features are subdivided into the following classes:

**First Order Features(18)**

InterquartileRange

Skewness

Uniformity

Median

Energy

RobustMeanAbsoluteDeviation

MeanAbsoluteDeviation

TotalEnergy

Maximum

RootMeanSquared

90Percentile

Minimum

Entropy

Range

Variance

10Percentile

Kurtosis

Mean

**Shape Features (13)**

Maximum3DDiameter

Maximum2DDiameterSlice

Sphericity

MinorAxis

Elongation

SurfaceVolumeRatio

Volume

MajorAxis

SurfaceArea

Flatness

LeastAxis

Maximum2DDiameterColumn

Maximum2DDiameterRow

**Gray Level Dependence Matrix (GLDM) Features (14)**

GrayLevelVariance

HighGrayLevelEmphasis

DependenceEntropy

DependenceNonUniformity

GrayLevelNonUniformity

SmallDependenceEmphasis

SmallDependenceHighGrayLevelEmphasis

DependenceNonUniformityNormalized

LargeDependenceEmphasis

LargeDependenceLowGrayLevelEmphasis

DependenceVariance

LargeDependenceHighGrayLevelEmphasis

SmallDependenceLowGrayLevelEmphasis

LowGrayLevelEmphasis

**Gray Level Co-occurrence Matrix (GLCM) Features (23)**

JointAverage

SumAverage

JointEntropy

ClusterShade

MaximumProbability

Idmn

JointEnergy

Contrast

DifferenceEntropy

InverseVariance

DifferenceVariance

Idn

Idm

Correlation

Autocorrelation

SumEntropy

SumSquares

ClusterProminence

Imc2

Imc1

DifferenceAverage

Id

ClusterTendency

**Gray Level Run Length Matrix (GLRLM) Features (16)**

ShortRunLowGrayLevelEmphasis

GrayLevelVariance

LowGrayLevelRunEmphasis

GrayLevelNonUniformityNormalized

RunVariance

GrayLevelNonUniformity

LongRunEmphasis

ShortRunHighGrayLevelEmphasis

RunLengthNonUniformity

ShortRunEmphasis

LongRunHighGrayLevelEmphasis

RunPercentage

LongRunLowGrayLevelEmphasis

RunEntropy

HighGrayLevelRunEmphasis

RunLengthNonUniformityNormalized

**Gray Level Size Zone Matrix (GLSZM) Features (16)**

GrayLevelVariance

ZoneVariance

GrayLevelNonUniformityNormalized

SizeZoneNonUniformityNormalized

SizeZoneNonUniformity

GrayLevelNonUniformity

LargeAreaEmphasis

SmallAreaHighGrayLevelEmphasis

ZonePercentage

LargeAreaLowGrayLevelEmphasis

LargeAreaHighGrayLevelEmphasis

HighGrayLevelZoneEmphasis

SmallAreaEmphasis

LowGrayLevelZoneEmphasis

ZoneEntropy

SmallAreaLowGrayLevelEmphasis

**Neighbouring Gray Tone Difference Matrix (NGTDM) Features (5)**

Coarseness

Complexity

Strength

Contrast

Busyness
